# Proton Pump Inhibitor Use for Gastroprotection and Stress Ulcer Prophylaxis Does Not Increase the Risk of *Clostridioides difficile* Infection or Pneumonia: A Systematic Review and Meta-Analysis of RCTs

**DOI:** 10.3390/jcm15072617

**Published:** 2026-03-29

**Authors:** Mohamed A. Omar, Marcel Katrib, Rahul Shekhar, David Maundu, Abu Baker Sheikh, Jane Gitau, Nathan Tofteland

**Affiliations:** 1 Department of Internal Medicine, University of Kansas School of Medicine-Wichita, Wichita, KS 67214, USAmkatrib@kumc.edu (M.K.);; 2 Department of Internal Medicine/Pediatrics, University of Kansas School of Medicine-Wichita, Wichita, KS 67214, USA; 3 Department of Internal Medicine, University of New Mexico, Albuquerque, NM 87106, USA

**Keywords:** *Clostridioides difficile* infection, proton pump inhibitors, PPI, gastrointestinal bleeding

## Abstract

**Background:** Proton pump inhibitors (PPIs) are widely used to prevent acid-related complications, yet concerns persist about infectious harm. Observational studies have linked PPIs to *Clostridioides difficile* infection (CDI) and pneumonia whereas randomized controlled trials (RCTs) consistently show reductions in upper gastrointestinal bleeding. We therefore conducted a systematic review and meta-analysis restricted to randomized controlled trials to evaluate whether PPIs increase the risk of CDI, and to assess pneumonia and gastrointestinal bleeding to contextualize net clinical benefit. **Methods:** A comprehensive search of randomized controlled trials (RCTs) was conducted using several databases including PubMed, Embase, Cochrane Central Register of Controlled Trials (CENTRAL) and SCOPUS until July 2025. All published English-language RCTs that met the inclusion criteria were included. Random-effects models were utilized to calculate pooled odds ratios (ORs) with 95% confidence intervals. The risk of bias was assessed using the Cochrane Risk of Bias 2.0 Tool, and heterogeneity was quantified using I^2^ statistics. Analysis was performed using STATA version 18 and RevMan 5.3. **Results:** Across eight RCTs (n = 30,019), PPIs did not increase C. difficile infection versus placebo (OR 1.29, 95% CI 0.82–2.02; *p* = 0.27; I^2^ = 16%) with leave-one-out (LOO) analyses showing stable estimates. In six trials reporting pneumonia, there was no significant difference between groups (OR 1.00, 95% CI 0.92–1.09; *p* = 0.99; I^2^ = 0%). For clinically important upper GI bleeding (seven trials), PPIs were associated with a statistically significant lower risk when compared to placebo (OR 0.51, 95% CI 0.27–0.94; *p* = 0.03; I^2^ = 56%). **Conclusions:** Across randomized trials with follow-up ranging from 30 days to 3 years, PPI prophylaxis significantly reduced upper gastrointestinal bleeding without increasing the risk of CDI or pneumonia. These findings support the use of PPIs for prophylaxis when clinically indicated, while recognizing that larger trials are needed to better assess rare adverse events.

## 1. Introduction

Proton pump inhibitors (PPIs) are widely used as first-line agents for acid suppression and the prevention of gastrointestinal ulcers in both inpatient and outpatient settings. In the United States, up to 8.6% of the population have active prescriptions for PPIs, with millions more using over-the-counter formulations [1]. PPIs account for approximately $10 billion in annual healthcare expenditures, and estimates suggest that up to 15% of Americans have used a PPI within the past year [2]. This widespread use underscores the importance of understanding both the therapeutic benefits and potential risks associated with PPI therapy.

*Clostridioides difficile* has long been a common cause of healthcare-associated infections affecting approximately 600,000 individuals annually in the United States and resulting in an estimated 44,500 deaths [3]. Its manifestations range from self-limiting diarrhea to life-threatening pseudomembranous colitis [4]. According to CDC surveillance data, the crude incidence rate is 116.1 cases per 100,000 persons, with higher rates observed in older adults and women [5]. The economic burden is substantial, with *Clostridioides difficile* infection (CDI) contributing up to $5.4 billion annually in healthcare costs, primarily driven by hospitalizations [3]. Prior studies have linked CDI to PPI exposure, raising concerns about the safety of these widely used medications.

Proposed mechanisms linking PPI use to CDI include gastric acid suppression, which impairs the stomach’s natural barrier against ingested pathogens. Experimental models have shown that reduced gastric acidity significantly increases the survival of ingested bacterial pathogens, including C. difficile spores [6]. Additionally, PPIs may contribute to alterations in the gut microbiome, reducing microbial diversity and disrupting colonization resistance. Studies have demonstrated that PPI users exhibit increased abundance of potentially pathogenic bacteria such as *Enterococcus*, *Streptococcus*, and *Escherichia coli*, along with a decrease in beneficial taxa [7]. Furthermore, recent bioreactor studies suggest that the increased risk of CDI is primarily driven by pH-mediated changes in the gut environment rather than direct antimicrobial effects of PPIs [8,9,10]. While these mechanisms are biologically plausible, much of the supporting evidence comes from observational studies, which are prone to confounding and may overestimate the true risk.

To address this limitation, we conducted a systematic review and meta-analysis restricted to randomized controlled trials (RCTs) to evaluate whether PPIs increase the risk of CDI. We also examined the incidence of pneumonia and gastrointestinal bleeding to contextualize the overall clinical benefit of PPI therapy. By focusing exclusively on RCTs, this analysis aims to provide more robust and reliable estimates of both efficacy and safety compared to those derived from observational data.

## 2. Methods

### 2.1. Study Design

This meta-analysis was conducted in accordance with the guidelines specified in the Preferred Reporting Items for Systematic Reviews and Meta-Analyses (PRISMA) Statement [11]. As Supplementary Material you can find the PRISMA checklist [12]. As this study constitutes a meta-analysis, ethical approval and informed consent were not required.

### 2.2. Identification of Studies

A comprehensive search of randomized controlled trials (RCTs) was conducted using several databases including PubMed, Embase, Cochrane Central Register of Controlled Trials (CENTRAL) and Scopus. The search period extended until July 2025 and included all published English-language RCTs that met the inclusion criteria. A manual citation search of references from retrieved articles was also performed to identify any relevant studies that were missed through database search.

### 2.3. Inclusion and Exclusion Criteria

Only RCTs comparing the risk of developing CDI in patients receiving a PPI versus placebo were included. Studies were eligible if they

1. Included adult participants (≥18 years) receiving a PPI;
2. Reported CDI along with at least one other outcome of interest: either pneumonia or upper gastrointestinal bleeding (or both).

Studies were excluded if they

1. Were quasi-randomized or non-randomized;
2. Did not report CDI as an outcome;
3. Lacked adequate data for extraction or analysis.

### 2.4. Data Extraction and Quality Assessment

Two independent reviewers (MO and DM) screened titles and abstracts for eligibility, followed by full-text assessments. Disagreements were resolved through discussion and by an independent third reviewer (RS). Data on study design, patient demographics, intervention and control treatments, and primary outcomes were extracted.

**Figure 1:**
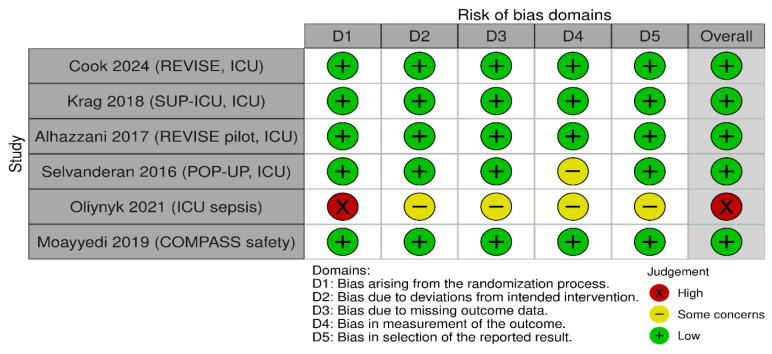
Risk of bias assessment for development of pneumonia when treated with PPI [14,15,16,17,18,19].

**Figure 2:**
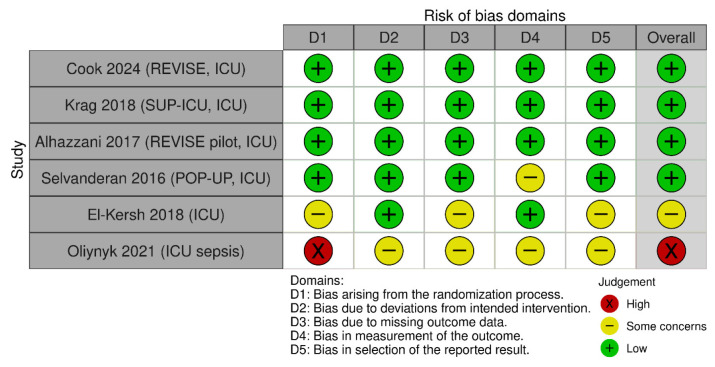
Risk of bias for clinically important upper gastrointestinal bleeding [14,15,16,17,18,19].

**Figure 3:**
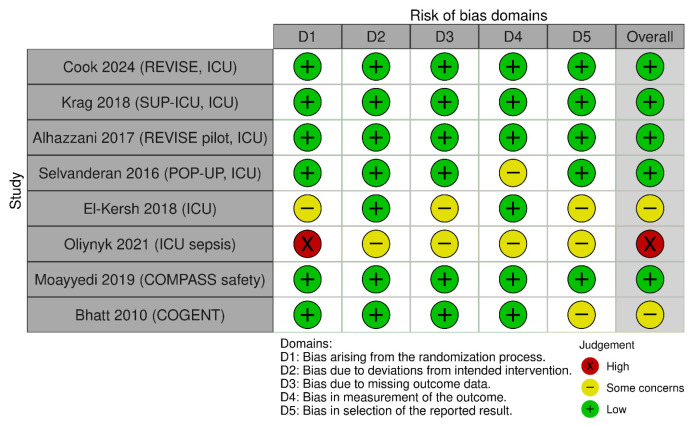
Risk of bias assessment for development of CDI [14,15,16,17,18,19,20,21].

The methodological quality and risk of bias of the included studies were assessed using the Cochrane Risk of Bias Tool (RoB 2.0) [13]. Domains evaluated included random sequence generation, allocation concealment, blinding, incomplete outcome data and selective reporting (Figure 1, Figure 2 and Figure 3).

### 2.5. Outcomes

The primary outcome for this study was

1. *Clostridioides difficile* infection while on treatment with the proton pump inhibitor and placebo.

The secondary outcomes of interest were:

1. Pneumonia, defined as either community-acquired pneumonia or ventilator-acquired pneumonia for patients who were in the ICU while on treatment;
2. Gastrointestinal bleeding, defined as the incidence of clinically important GI bleeding while the patient was on treatment.

### 2.6. Data Synthesis and Analysis

In individual trials, we estimated odds ratios (ORs) for dichotomous outcomes and mean differences for continuous outcomes, together with their 95% confidence intervals (CIs). We evaluated the variability in intervention effects across primary studies by employing the χ2 test (Cochran Q) and the I2 statistic. Pooled estimates were calculated using the DerSimonian and Laird random-effects model [22]. We interpreted I^2^ values of 25%, 50%, and 75% as representing low, moderate, and high heterogeneity, respectively [23].

Sensitivity analyses were conducted using the leave-one-out method for CDI, pneumonia, and gastrointestinal bleeding to evaluate the impact of individual studies on the overall effect estimate. Given that the number of studies included was less than 10, assessment of publication bias using funnel plots was deferred. We rated the quality of evidence according to the Grades of Recommendation, Assessment, Development and Evaluation approach (GRADE) [24], and findings are shown in Table 1.

### 2.7. Statistical Analysis

Sensitivity analyses were conducted using the leave-one-out method to evaluate the robustness of pooled estimates. We performed leave-one-out sensitivity analyses using Stata version 18 (StataCorp LP, StataCorp, College Station, TX, USA). The remaining analyses were conducted with RevMan 5.3 (The Cochrane Collaboration, Copenhagen, Denmark).

## 3. Results

**Figure 4:**
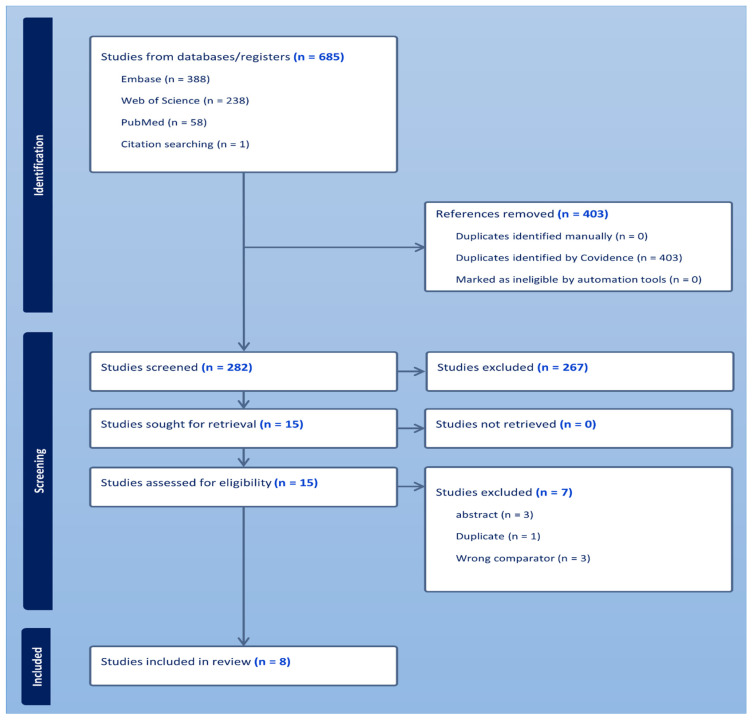
Prisma flow of included studies.

The study selection process is demonstrated in Figure 4. A total of 688 records were retrieved by searching the different databases and trial registers. After 403 duplicates were removed, an additional 274 records were removed after the titles and abstracts were screened. After full text screening, a total of eight randomized controlled trials encompassing 30,019 participants were included in this meta-analysis [14,15,16,17,18,19,20,21].

All publications were RCTs, five of which were multicenter studies. Six studies were based in ICU settings [15,16,17,18,19,21] whereas the remaining two included both inpatient and outpatient healthcare settings [14,20]. The authors compared the effect of proton pump inhibitors to placebo, and the articles included used different dosages of PPIs and various treatment administration and durations. The basic characteristics of the included studies are detailed in Table 2.

### 3.1. Primary Outcome

#### Risk of Developing CDI on PPIs Versus Placebo

**Figure 5:**
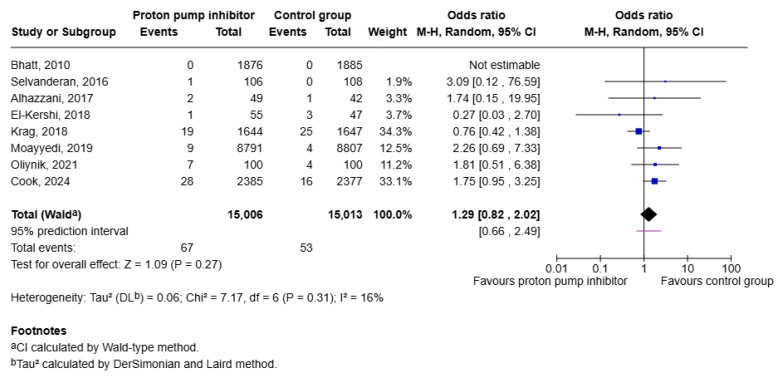
Forest plot showing risk of CDI in patients taking PPIs versus placebo [14,15,16,17,18,19,20,21].

Eight RCTs totaling 30,019 patients (15,006 in the intervention and 15,013 in the placebo group) evaluated the rates of CDI in participants receiving PPI therapy compared to placebo. No significant difference in risk of CDI was observed between the two study groups [OR 1.29 (95 CI 0.82, 2.02); *p* = 0.27]. Low heterogeneity was noted among the studies (I^2^ = 16%; Tau = 0.06) (Figure 5).

**Figure 6:**
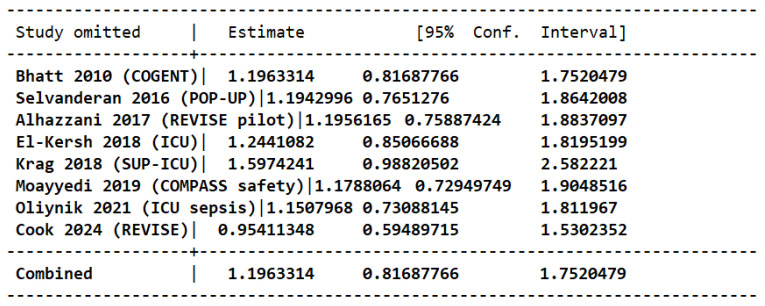
Leave-one-out analysis for risk of CDI in patients taking PPIs versus placebo [14,15,16,17,18,19,20,21].

The overall effect was confirmed by performing leave-one-out sensitivity analysis which demonstrated a consistent magnitude of effect (Figure 6).

### 3.2. Secondary Outcomes

#### 3.2.1. Risk of Developing Pneumonia on PPIs Versus Placebo

**Figure 7:**
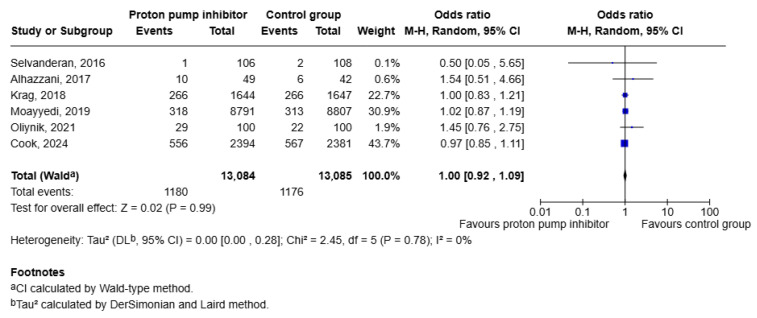
Forest plot showing risk of pneumonia in patients taking PPIs versus placebo [14,15,16,17,18,19].

Pneumonia was described in six of the eight trials included in this study [14,15,16,17,18,19]. Analysis of the pooled estimate revealed no significant difference in the incidence of pneumonia between patients receiving PPI therapy compared to placebo [OR 1.00 (95 CI: 0.92, 1.09); *p* = 0.99]. No heterogeneity was noted in this outcome, signifying no variability in this finding among the different studies (I^2^ = 0%; Tau = 0.00) (Figure 7).

**Figure 8:**
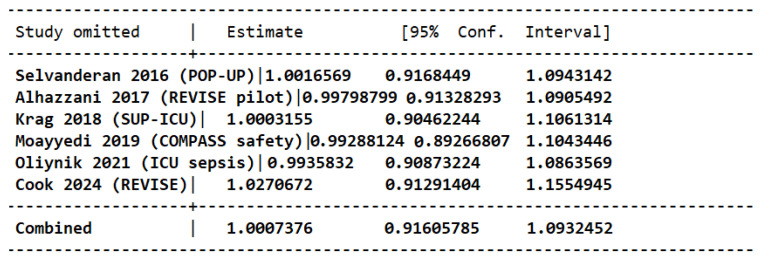
Leave-one-out analysis for risk of pneumonia in patients taking PPIs versus placebo [14,15,16,17,18,19].

Stability was evaluated by sensitivity analysis using the leave-one-out approach (Figure 8); direction and magnitude of effect remained consistent in all iterations.

#### 3.2.2. Risk of Clinically Important Gastrointestinal Bleeding

**Figure 9:**
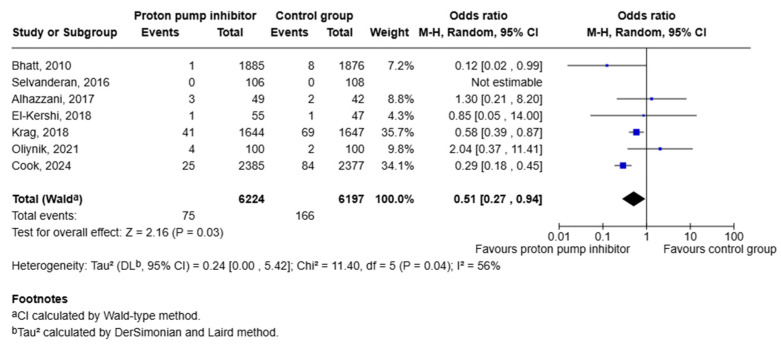
Forest plot showing risk of upper GI bleeding in patients taking PPIs versus placebo [15,16,17,18,19,20,21].

Data from seven studies were pooled to establish the risk of developing gastrointestinal bleeding when treated with PPIs as opposed to placebo [15,16,17,18,19,20,21]. Our analysis found a statistically significant lower risk of gastrointestinal bleeding with PPI therapy when compared to placebo [OR 0.51 (95% CI: 0.27, 0.94); *p* = 0.03]. Moderate heterogeneity was noted in this analysis (I^2^ = 56%; Tau = 0.24) (Figure 9).

**Figure 10:**
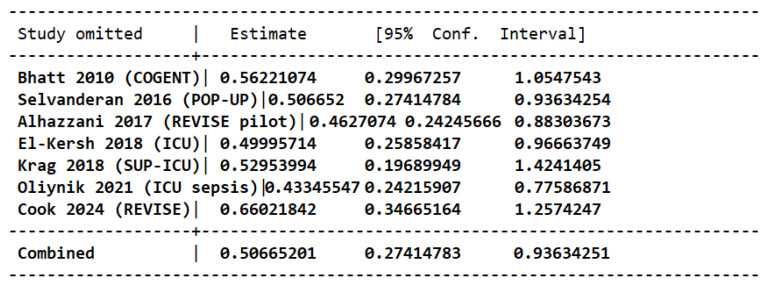
Leave-one-out analysis for risk of upper GI bleeding in patients taking PPIs versus placebo [15,16,17,18,19,20,21].

Leave-one-out sensitivity showed stable direction of effect (RRs 0.44–0.67). Excluding the largest trial by Cook et al. attenuated the precision (RR 0.67, 95% CI 0.36–1.27), but no single study reversed the protective association of PPIs against upper-GI bleeding [19] (Figure 10).

## 4. Discussion

In this meta-analysis of randomized controlled trials including more than 30,000 patients, PPI prophylaxis significantly reduced clinically important upper gastrointestinal bleeding without increasing the risk of CDI or pneumonia. These findings provide high-certainty evidence that PPIs remain effective for bleeding prevention, while suggested infectious harms have not been demonstrated in randomized data with PPI exposure ranging from less than 30 days to 3 years [14,18]. The net clinical impact therefore depends more on baseline bleeding risk than on concerns about CDI or pneumonia.

Despite biologically plausible mechanisms linking PPIs to infection including impaired gastric bacteriostasis and alterations of the gut microbiome, our pooled estimates did not show increased risks of CDI or pneumonia [9,10]. Importantly, CDI was infrequent in the included RCTs, and most cases occurred in the context of antibiotic exposure rather than acid suppression alone. From a clinical perspective, although the point estimate suggests a possible 29% relative increase in CDI, the wide confidence interval ranging from a potential 18% risk reduction to a 102% increased risk reflects substantial uncertainty. The low absolute event rates across studies (67 vs. 53 cases; an absolute risk difference of 14 CDI cases among >30,000 total participants) also suggest that even if a risk increase exists, the absolute clinical impact is likely small in this trial-based population. Additionally, heterogeneity was low (I^2^ = 16%), reinforcing that the lack of statistical significance is consistent across studies and is not driven by outliers. Overall, current RCT evidence does not demonstrate a statistically or clinically meaningful increase in CDI risk with PPIs, though the direction of the effect warrants continued attention in future large-scale RCTs. Prior meta-analyses of observational studies reported relative risks for CDI ranging from 1.26 to 2.34, but these likely reflect residual confounding [25,26,27]. Observational designs are inherently vulnerable to confounding by indication, differential exposure misclassification, variability in diagnostic criteria, and unmeasured covariates such as illness severity, antimicrobial exposure, and comorbidity burden, all of which limit causal inference. In contrast, our meta-analysis, restricted to randomized controlled trials where treatment allocation is balanced and major confounders are controlled by design, did not demonstrate an increased risk of CDI with PPI therapy. This supports the interpretation that the associations detected in prior observational research are more consistent with residual bias than with a true causal effect [25,26,27]. The consistency of our null estimate with large, contemporary trials such as REVISE, which found no differences in CDI or ventilator-associated pneumonia between pantoprazole and placebo, strengthens the idea that PPIs are not major independent drivers of infection risk in hospitalized or ICU patients [19]. Notably, in the COMPASS randomized placebo-controlled trial, pantoprazole use was associated with a small but statistically significant increase in non-CDI enteric infections compared with placebo (1.4% vs. 1.0%; odds ratio 1.33, 95% CI 1.01–1.75), with no statistically significant differences observed in other prespecified safety outcomes [14].

The reduction in GI bleeding observed in our pooled analysis is clinically consistent with individual trial data. In REVISE, pantoprazole reduced clinically important bleeding from 3.5% to 1.0% without affecting mortality, while in the COGENT trial, omeprazole substantially reduced GI events among patients on dual antiplatelet therapy [19,20]. However, in lower-risk ICU populations with systematic early enteral feeding, such as POP-UP and a trial of pantoprazole plus enteral nutrition, bleeding rates were negligible, and prophylaxis provided no measurable benefit [15,21]. These findings suggest that absolute benefit varies considerably with baseline risk: prophylaxis has clear value in high-risk groups but is unlikely to provide benefit in patients already protected by early feeding and low baseline bleeding incidence.

Our results align with guideline recommendations and a 2020 network meta-analysis, which concluded that acid suppression prevents bleeding in the ICU without affecting mortality [28]. What our analysis adds is high-quality evidence that CDI and pneumonia risks are not significantly increased in randomized populations, thereby narrowing the uncertainty that previously limited guideline strength. Clinically, this supports a risk-stratified approach: PPIs are appropriate in patients at high bleeding risk (e.g., invasive ventilation, coagulopathy, or dual antiplatelet therapy), while routine use in low-risk ICU or ambulatory patients is unlikely to provide meaningful benefit.

This meta-analysis has several notable strengths. By restricting inclusion to randomized controlled trials, we minimized the confounding and bias inherent in observational studies and provided higher certainty estimates of both benefit and harm. The large, pooled sample of over 30,000 patients enabled the evaluation of uncommon outcomes such as CDI, while the inclusion of diverse trial populations across ICU and non-ICU settings enhances generalizability. Rigorous methodology was applied, including duplicate screening, standardized risk-of-bias assessment, sensitivity analyses, and GRADE evaluation of evidence certainty.

Several limitations should also be acknowledged. While our meta-analysis provides an overall estimate of infection risk associated with PPI use, there is meaningful clinical heterogeneity across the included trials. In particular, the largest numerical contributions to the CDI outcome came from the REVISE and SUP-ICU trials, which enrolled critically ill ICU patients receiving IV pantoprazole 40 mg for stress ulcer prophylaxis [17,19]. Notably, these two trials showed opposing directions of effect for CDI counts, underscoring how baseline infection risk, concomitant antibiotic exposure, and ICU-specific factors may influence observed event rates. CDI was relatively rare and often reported as a secondary outcome, which limited statistical power and precision even when the total sample size was large. This reduced the ability to definitively exclude a modest increase in CDI risk. For gastrointestinal bleeding, moderate heterogeneity was observed (I^2^ = 56%), and event rates differed substantially by baseline bleeding risk and clinical context, limiting the applicability of pooled estimates to lower-risk populations. In ICU stress-ulcer prophylaxis trials, bleeding risk is strongly shaped by the severity of illness, mechanical ventilation, coagulopathy, and concurrent anticoagulant exposure, whereas ambulatory gastroprotection trials involve different competing risks and varying follow-up intervals, which contributed to variability in observed effects. Although the overall sample size was large, an assessment of long-term adverse outcomes such as fractures, kidney disease, or dementia was not conducted. Finally, the modest number of included trials prevented formal evaluation of publication bias.

Our RCT-only meta-analysis addresses a critical gap left by prior observational evidence by providing high-quality trial data on the safety and efficacy of PPIs in hospitalized patients. We demonstrate that PPIs reliably reduce clinically important gastrointestinal bleeding without increasing the risk of CDI or pneumonia, thereby narrowing the uncertainty that has long surrounded their use. By showing that infectious harms are not evident in randomized populations, this study refines the balance of benefits and risks and supports a risk-stratified approach to prophylaxis, reserving PPIs for patients at greatest bleeding risk. These findings add clarity to a field dominated by conflicting data and establish a firmer foundation for guideline recommendations, while underscoring the need for future trials with longer follow-up to evaluate rare or delayed adverse effects.

## 5. Conclusions

This review of evidence from randomized studies suggests that PPIs deliver clear benefit with no demonstrable increase in CDI or pneumonia at the population level across both short- and long-term use. Furthermore, the absolute excess enteric infection risk appears small. Thus, considering previously unresolved safety questions, our synthesis supports the safe use of PPIs in both the acute and ambulatory setting with no increased risk of C. difficile infection.

## Figures and Tables

**Table 1:** GRADE assessment of certainty of evidence of included studies.

**Question:** Does the use of proton pump inhibitors increase the risk of developing Clostridioides difficile infection												
**Setting:** Both ICU and outpatient settings												
**Certainty Assessment**							**№ of Patients**		**Effect**		**Certainty**	**Importance**
**№ of Studies**	**Study Design**	**Risk of Bias**	**Inconsistency**	**Indirectness**	**Imprecision**	**Other Considerations**	**Proton Pump Inhibitors**	**Placebo**	**Relative** **(95% CI)**	**Absolute** **(95% CI)**		
**Risk of *Clostridioides difficile* infection**												
8	Randomised trials	not serious	serious ^a^	not serious	serious ^b^	none	67/15,006 (0.4%)	53/15,013 (0.4%)	**OR 1.29** (0.82 to 2.02)	1 more per 1000 (from 1 fewer to 4 more)	⨁⨁◯◯ Low ^a,b^	IMPORTANT
**Risk of developing pneumonia with proton pump inhibitors**												
6	Randomised trials	not serious	not serious	not serious	serious^c^	none	1180/13,084 (9.0%)	1176/13,085 (9.0%)	**RR 1.00** (0.92 to 1.09)	0 fewer per 1000 (from 7 fewer to 8 more)	⨁⨁⨁◯ Moderate ^c^	IMPORTANT
**Clinically important upper GI bleeding**												
6	Randomised trials	not serious	not serious	not serious	not serious	none	74/4339 (1.7%)	158/4321 (3.7%)	**RR 0.51** (0.27 to 0.94)	18 fewer per 1000 (from 27 fewer to 2 fewer)	⨁⨁⨁⨁ High	IMPORTANT

**CI:** confidence interval; **OR:** odds ratio; **RR:** risk ratio. a. very rare events and small trials vary; b. Very low event rates; c. event rates are modest with wide confidence intervals.

**Table 2:** Basic characteristics of included studies.

Study Name	Location	Setting	Intervention	Comparator	Total Sample Size	Indication	Baseline Demographics		Outcomes of Interest	Length of Follow-Up
							Intervention Group	Comparator Group		
Bhatt 2010 (COGENT) [20]	International	Both acute and outpatient healthcare settings	20 mg Omeprazole (oral)	Placebo	3761	Gastroprotection and bleeding prophylaxis in patients with dual antiplatelet therapy	-Median age: 68.5 years -Males: 66.9%	-Median age: 68.7 years -Males: 69.5%	- CDI -Upper GI bleeding	-Median follow-up 106 days (IQR 55–166), max 341 days
Selvanderan 2016 (POP-UP) [15]	Australia	Mixed medical–surgical ICU	40 mg Pantoprazole (intravenous)	Placebo	214	Stress ulcer prophylaxis	-Age (mean ± SD): 52 ± 18 years -Males: 64%	-Age (mean ± SD): 52 ± 17 years -Males: 67%	-CDI -Pneumonia -Upper GI bleeding	-Outcomes were assessed throughout the ICU stay, with pneumonia additionally monitored up to 7 days after stopping the study drug and C. difficile infection monitored until hospital discharge.
Alhazzani 2017 (REVISE pilot) [16]	Canada, Saudi Arabia, and Australia	ICUs	40 mg Pantoprazole (intravenous)	Placebo	91	Stress ulcer prophylaxis	-Median age: 61.8 years -Males: 55.1%	-Median age: 55.3 years -Males: 59.5%	-CDI -Pneumonia -Upper GI bleeding	-All outcomes were monitored throughout the ICU stay, with C. difficile followed until hospital discharge.
El-Kersh 2018 [21]	USA	ICUs	40 mg Pantoprazole (intravenous)	Placebo	102	Stress ulcer prophylaxis	-Median age: 62 years -Males: 55%	-Median age: 58 years -Males: 60%	-CDI -Upper GI bleeding	-Patients were followed until ICU discharge or cessation of enteral nutrition with transition to oral feeding.
Krag 2018 (SUP-ICU) [17]	Denmark, Finland, Netherlands, Norway, Switzerland, and the UK	ICUs	40 mg Pantoprazole (intravenous)	Placebo	3291	Stress ulcer prophylaxis	-Median age: 67 years -Males: 63%	-Median age: 67 years -Males: 65%	-CDI -Pneumonia -Upper GI bleeding	-Outcomes were assessed during the ICU stay.
Moayyedi 2019 (COMPASS safety) [14]	International	Outpatient and inpatient healthcare settings	40 mg Pantoprazole (oral)	Placebo	17,598	Bleeding prophylaxis in patients with stable atherosclerotic disease on aspirin/rivaroxaban	-Age (mean ± SD): 67.6 ± 8.1 years -Males: 78%	-Age (mean ± SD): 67.7 ± 8.1 years -Males: 79%	-CDI -Pneumonia	-All outcomes were assessed at structured follow-up visits every 6 months for a median of 3.01 years.
Oliynyk 2020 [18]	Poland	Neurotrauma ICU	0.2 mg/kg Omeprazole (intravenous)	Placebo	200	Stress ulcer prophylaxis	-Age (mean ± SD): 48.6 ± 7.6 years	-Age (mean ± SD): 46.3 ± 6.8 years	-CDI -Pneumonia -Upper GI bleeding	-Patients were monitored for one month.
Cook 2024 (REVISE) [19]	International	ICUs	40 mg Pantoprazole (intravenous)	Placebo	4821	Stress ulcer prophylaxis	-Age (mean ± SD): 58.2 ± 16.4 years -Males: 63.5%	-Age (mean ± SD): 58.3 ± 16.4 years -Males: 63.8%	-CDI -Pneumonia -Upper GI bleeding	-Upper GI bleeding was followed for 90 days after randomization. Pneumonia was assessed during the ICU stay, and C. difficile infection was assessed during the hospital stay.

## Data Availability

No new data were created or analyzed in this study.

## Supplementary Materials

The following supporting information can be downloaded at: https://www.mdpi.com/article/10.3390/jcm15072617/s1, PRISMA checklist: PRISMA 2020 for Abstract Checklist.
